# Evaluating the impact of healthcare interventions using routine data

**DOI:** 10.1136/bmj.l2239

**Published:** 2019-06-20

**Authors:** Geraldine M Clarke, Stefano Conti, Arne T Wolters, Adam Steventon

**Affiliations:** 1 The Health Foundation, London, UK; 2 NHS England and NHS Improvement, London, UK

**What you need to know**

Assessing the impact of healthcare interventions is critical to inform future decisions
Compare observed outcomes with what you would have expected if the intervention had not been implemented
A wide range of routinely collected data is available for the evaluation of healthcare interventions

Interventions to transform the delivery of health and social care are being implemented widely, such as those linked to Accountable Care Organizations in the United States,^1^ or to integrated care systems in the UK.^2^ Assessing the impact of these health interventions enables healthcare teams to learn and to improve services, and can inform future policy.^3^ However, some healthcare interventions are implemented without high quality evaluation, in ways that require onerous data collection, or may not be evaluated at all.^4^

A range of routinely collected administrative and clinically generated healthcare data could be used to evaluate the impact of interventions to improve care. However, there is a lack of guidance as to where relevant routine data can be found or accessed and how they can be linked to other data. A diverse array of methodological literature can also make it hard to understand which methods to apply to analyse the data. This article provides an introduction to help clinicians, commissioners, and other healthcare professionals wishing to commission, interpret, or perform an impact evaluation of a health intervention. We highlight what to consider and discuss key concepts relating to design, analysis, implementation, and interpretation.

## What are interventions, impacts, and impact evaluations?

A health intervention is a combination of activities or strategies designed to assess, improve, maintain, promote, or modify health among individuals or an entire population. Interventions can include educational or care programmes, policy changes, environmental improvements, or health promotion campaigns. Interventions that include multiple independent or interacting components are referred to as complex.^5^ The impact of any intervention is likely to be shaped as much by the context (eg, communities, work places, homes, schools, or hospitals) in which it is delivered, as the details of the intervention itself.^6^ ^7^ ^8^ ^9^

An impact is a positive or negative, direct or indirect, intended or unintended change produced by an intervention. An impact evaluation is a systematic and empirical investigation of the effects of an intervention; it assesses to what extent the outcomes experienced by affected individuals were caused by the intervention in question, and what can be attributed to other factors such as other interventions, socioeconomic trends, and political or environmental conditions. Evaluations can be categorised as formative or summative (table 1).

**Table 1:** Impact evaluations

Formative	Summative	Examples
Conducted during the development or implementation of an intervention	Conducted after the intervention’s completion, or at the end of a programme cycle	A formative evaluation of the Whole Systems Integrated Care (WSIC) programme, aimed at integrating health and social care in London, found that difficulties in establishing data sharing and information governance, and differences in professional culture were hampering efforts to implement change^10^
Aims to fine tune or reorient the intervention	Aims to render judgment, or make decisions about the future of the intervention	A summative impact evaluation of an NHS new care model vanguard initiative found that care home residents in Nottinghamshire who received enhanced support had substantially fewer attendances at emergency departments and fewer emergency admissions than a matched control group.^13^ This evidence supported the decision by the NHS to roll out the Enhanced Health in Care Homes Model across the country.^2^

Approaches such as the Plan, Do, Study, Act cycle^11^, which is part of the Model for Improvement, a commonly used tool to test and understand small changes in quality improvement work^12^ may be used to undertake formative evaluation.

With either type of evaluation, it is important to be realistic about how long it will take to see the intended effects. Assessment that takes place too soon risks incorrectly concluding that there was no impact. This might lead stakeholders to question the value of the intervention, when later assessment might have shown a different picture. For example, in a small case study of cost savings from proactively managing high risk patients, the costs of healthcare for the eligible intervention population initially increased compared with the comparison population, but after six months were consistently lower.^14^

This article focuses on impact evaluation, but this can only ever address a fraction of questions.^15^ Much more can be accomplished if it is supplemented with other qualitative and quantitative methods, including process evaluation. This provides context, assesses how the intervention was implemented, identifies any emerging unintended pathways, and is important for understanding what happened in practice and for identifying areas for improvement.^16^ The economic evaluation of healthcare interventions is also important for healthcare decision making, especially with ongoing financial pressures on health services.^17^

## What are the right evaluation questions?

An effective impact evaluation begins with the formulation of one or more clear questions driven by the purpose of the evaluation and what you and your stakeholders want to learn. For example, *“What is the impact of case management on patients’ experience of care?*”

Formulate your evaluation questions using your understanding of the idea behind your intervention, the implementation challenges, and your knowledge of what data are available to measure outcomes. Review your theory of change or logic model^21^ ^22^ to understand what inputs and activities were planned, and what outcomes were expected and when. Once you have understood the intended causal pathway, consider the practical aspects of implementation, which include the barriers to change, unexpected changes by recipients or providers, and other influences not previously accounted for. Patient and public involvement (PPI) in setting the right question is strongly recommended for additional insights and meaningful results. For example, if evaluating the impact of case management, you could engage patients to understand what outcomes matter most to them. Healthcare leaders may emphasise metrics such as emergency admissions, but other aspects such as the experience of care might matter more to patients.^5^ ^23^

## What methods can be used to perform an impact evaluation?

*Randomised control designs*, where individuals are randomly selected to receive either an intervention or a control treatment, are often referred to as the “gold standard” of causal impact evaluation.^24^ In large enough samples, the process of randomisation ensures a balance in observed and unobserved characteristics between treatment and control groups. However, while often suitable for assessing, for example, the safety and efficacy of medicines, these designs may be impractical, unethical, or irrelevant when assessing the impact of complex changes to health service delivery.

*Observational studies* are an alternative approach to estimate causal effects. They use the natural, or unplanned, variation in a population in relation to the exposure to an intervention, or the factors that affect its outcomes, to remove the consequences of a non-randomised selection process.^25^ The idea is to mimic a randomised control design by ensuring treated and control groups are equivalent—at least in terms of *observed* characteristics. This can be achieved using a variety of well documented methods, including regression control and matching,^26^ eg, propensity scoring^27^ or genetic matching.^28^ If the matching is successful at producing such groups, and there are also no differences in *unobserved* characteristics, then it can be assumed that the control group outcomes are representative of those that the treated group would have experienced if nothing had changed, ie, the counterfactual. For example, an evaluation of alternative elective surgical interventions for primary total hip replacement on osteoarthritis patients in England and Wales used genetic matching to compare patients across three different prosthesis groups, and reported that the most prevalent type of hip replacement was the least cost effective.^29^

Assessing similarity is only possible in relation to *observed* characteristics, and matching can result in biased estimates if the groups differ in relation to *unobserved* variables that are predictive of the outcome (confounders). It is rarely possible to eliminate this possibility of bias when conducting observational studies, meaning that the interpretation of the findings must always be sensitive to the possibility that the differences in outcomes were caused by a factor other than the intervention. Methods that can help when selection is on unobserved characteristics include difference-in-difference,^30^ regression discontinuity,^31^ instrumental variables,^18^ or synthetic controls.^32^ Table 2 gives a summary of selected observational study designs.

**Table 2:** **Observational study designs for quantitative impact evaluation**

Method	Strengths and limitations
***Matching*** 33 Aims to find a subset of control group units (eg, individuals or hospitals) with similar characteristics to the intervention group units in the pre-intervention period. For example, impact of enhanced support in care homes in Rushcliffe, Nottinghamshire^13^	Can be combined with other methods, eg, difference-in-differences and regression. Enables straightforward comparison between intervention and control groups. Methods include propensity score matching and genetic matching
***Regression control*** 34 Refers to use of regression techniques to estimate association between an intervention and an outcome while holding the value of the other variables constant, thus adjusting for these variables	Can be beneficial to pre-process the data using matching in addition to regression control. This reduces the dependence of the estimated treatment effect on how the regression models are specified^35^
***Difference-in-differences (DiD*** *)* 30 Compares outcomes before and after an intervention in intervention and control group units. Controls for the effects of unobserved confounders that do not vary over time, eg, impact of hospital pay for performance on mortality in England^36^	Simple to implement and intuitive to interpret. Depends on the assumption that there are no unobserved differences between the intervention and control groups that vary over time, also referred to as the “parallel trends” assumption
***Synthetic controls*** 32 Typically used when an intervention affects a whole population (eg, region or hospital) for whom a well matched control group comprising whole control units is not available. Builds a “synthetic” control from a weighted average of the control group units, eg, impact of redesigning urgent and emergency care in Northumberland^37^	Allows for unobserved differences between the intervention and control groups to vary over time. The uncertainty of effect estimates is hard to quantify. Produces biased estimates over short pre-intervention periods
***Regression discontinuity design*** 31 Uses quasi-random variations in intervention exposure, eg, when patients are assigned to comparator groups depending on a threshold. Outcomes of patients just below the threshold are compared with those just above, eg, impact of statins on cholesterol by exploiting differences in statin prescribing^38^	There is usually a strong basis for assuming that patients close to either side of the threshold are similar. Because the method only uses data for patients near the threshold, the results might not be generalisable
***Interrupted time-series*** 39 Compares outcomes at multiple time points before and after an intervention (interruption) is implemented to determine whether the intervention has an effect that is statistically significantly greater than the underlying trend, eg, to examine the trends in diagnosis for people with dementia in the UK^40^	Ensures limited impact of selection bias and confounding as a result of population differences but does not generally control for confounding as a result of other interventions or events occurring at the same time as the intervention
***Instrumental variables*** 18 An instrumental variable is a variable that affects the outcome solely through the effect on whether the patient receives the treatment. An instrumental variable can be used to counteract issues of measurement error and unobserved confounders, eg, used to assess delivery of premature babies in dedicated *v* hospital intensive care units^19^	Explicitly addresses unmeasured confounding but conceptually difficult and easily misused. Identification of instrumental variables is not straightforward. Estimates are imprecise (large standard error), biased when sample size is small, and can be biased in large samples if assumptions are even slightly violated^20^

Observational studies are often referred to as natural (for natural or unplanned interventions), or quasi (for planned or intentional interventions) experiments. Natural experiments are discussed to evaluate population health interventions.^41^

## What’s wrong with a simple before-and-after study?

Before-and-after studies compare changes in outcomes for the *same* group of patients at a single time point before and after receiving an intervention without reference to a control group. These differ from interrupted time series studies, which compare changes in outcomes for *successive* groups of patients before and after receiving an intervention (the interruption).

Before-and-after studies are useful when it is not possible to include an unexposed control group, or for hypothesis generation. However, they are inherently susceptible to bias since changes observed may simply reflect regression to the mean (any changes in outcomes that might occur naturally in the absence of the intervention), or influences or secular trends unrelated to the intervention, eg, changes in the economic or political environment, or a heightened public awareness of issues.

For example, a before-and-after study of the impact of a care coordination service for older people tracked the hospital utilisation of the same patients before and after they were accepted into the service. They found that the service resulted in savings in hospital bed days and attendances at the emergency department.^42^ Reduced hospital utilisation could have reflected regression to the mean here rather than the effects of the intervention; for example, a patient could have had a specific health crisis before being invited to join the service and then reverted back to their previous state of health and hospital utilisation for reasons unconnected with the care coordination service.

Various tools are available to evaluate the risk of bias in non-randomised designs due to confounding and other potential biases.^43^ ^44^

## Where can I find suitable routine data?

Healthcare systems generate vast amounts of data as part of their routine operation. These datasets are often designed to support direct care, and for administrative purposes, rather than for research, and use of routinely collected data for evaluating changes in health service delivery is not without pitfalls. For example, any variation observed between geographical regions, providers, and sometimes individual clinicians may reflect real and important variations in the actual healthcare quality provided, but can also result from differences in measurement.^45^ However, routine data can be a rich source of information on a large group of patients with different conditions across different geographical regions. Often, data have been collected for many years, enabling construction of individual patient histories describing healthcare utilisation, diagnoses, comorbidities, prescription of medication, and other treatments.

Some of these data are collected centrally, across a wider system, and routinely shared for research and evaluation purposes, eg, secondary care data in England (Hospital Episode Statistics), or Medicare Claims data in the United States. Other sources, such as primary care data, are often collected at a more local level, but can be accessed through, or on behalf of, healthcare commissioners, provided the right information governance arrangements are in place. Pseudonymised records, where any identifying information is removed or replaced by an artificial identifier, are often used to support evaluation while maintaining patient confidentiality. See table 3 for commonly used routine datasets available in England.

**Table 3:** **Commonly used routine datasets available in the NHS in England**

Dataset	Dissemination and alternatives
***Hospital episode statistics (HES)***.^46^ HES is a database containing details of all admissions, accident and emergency attendances, and outpatient appointments at NHS England hospitals and NHS England funded treatment centres. Information captured includes clinical information about diagnoses and operations, patient demographics, geographical information, and administrative information such as the data and method of admissions and discharge	HES is available through the Data Access Request Service (DARS),^47^ a service provided by NHS Digital. Commissioners, providers in the NHS, and analytics teams working on their behalf, can also access hospital data directly via the Secondary Use Service (SUS).^48^ These data are very similar to HES, processed by NHS Digital, and are available for non-clinical uses, including research and planning health services
***Primary care data*** is collected by general practices. Although there is no national standard on how primary care data should be collected and/or reported, there are a limited number of commonly used software providers to record these data. Information captured includes clinical information about diagnoses, treatment, and prescriptions, patient demographics, geographical information, and administrative information on booking and attendance of appointments, and whether appointments relate to a telephone consultation, an in-practice appointment, or a home visit	Commissioners, and analytics teams working on their behalf, can work with an intermediary service called Data Service for Commissioning Regional Office to request access to anonymised patient level general practice data (possibly linked to SUS, described above) for the purpose of risk stratification, invoice validation, and to support commissioning. Anonymised UK primary care records for a representative sample of the population are available for public health research through, for instance, the Clinical Practice Research Datalink.^49^
***Mortality data*** 50 The Office for National Statistics (ONS) maintains a dataset of all registered deaths in England. These data can be linked to routine health data to record deaths that occur outside of hospital	ONS mortality data are routinely processed by NHS Digital, and can be linked to HES data. These data can be requested through the DARS service. When deaths occur in hospital this is typically recorded as part of discharge information
***The Mental Health Services Data Set (MHSDS)*** 51 contains record level data about the care of children, young people, and adults who are in contact with mental health, learning disabilities, or autism spectrum disorder services. These data cover data from April 2016	Like HES, MHSDS is available through the DARS service. Mental health data from before April 2016 have been recorded in the Mental Health Minimum Dataset also disseminated through NHS Digital

Healthcare records can often be linked across different sources as a single patient identifier is commonly used across a healthcare system, eg, the use of an NHS number in the UK. Using a common pseudonym across different data sources can support linkage of pseudonymised records. Linking into publicly available sources of administrative data and surveys can further enrich healthcare records. Commonly used administrative data available for UK populations include measures of GP practice quality and outcomes from the Quality and Outcomes Framework (QOF),^52^ deprivation, rurality, and demographics from the 2011 Census,^53^ and patient experience from the GP Patient Survey.^54^

## Are there any additional considerations?

It is essential to consider threats to validity when designing and evaluating an impact evaluation; validity relates to whether an evaluation is measuring what it is claiming to measure. See Rothman et al^55^ for further discussion.

*Internal validity* refers to whether the effects observed are due to the intervention and not some other confounding factor. Selection bias, which results from the way in which subjects are recruited, or from differing rates of participation due, for example, to age, gender, cultural or socioeconomic factors, is often a problem in non-randomised designs. Care must be taken to account for such biases when interpreting the results of an impact evaluation. Sensitivity analyses should be performed to provide reassurance regarding the plausibility of causal inferences.

*External validity* refers to the extent to which the results of a study can be generalised to other settings. Understanding the societal, economic, health system, and environmental context in which an intervention is delivered, and which makes its impact unique, is critical when interpreting the results of evaluations, and considering whether they apply to your setting.^56^ Descriptions of context should be as rich as possible.

Often, the impact of an intervention is likely to vary depending on the characteristics of patients. These can be usefully explored in subgroup analyses.^57^

Clear and transparent reporting using established guidelines (eg, STROBE^58^ or TREND^59^)to describe the intervention, study population, assignment of treatment, and control groups, and methods used to estimate impact should be followed. Limitations arising as a result of inherent biases, or validity, should be clearly acknowledged. 

Around the world, many interventions designed to improve health and healthcare are under way. An evaluation is an essential part of understanding what impact these changes are having, for whom and in what circumstances, and help inform future decisions about improvement and further roll out. There is no standard, ‘‘one size fits all’’ recipe for a good evaluation: it must be tailored to the project at hand. Understanding the overarching principles and standards is the first step towards a good evaluation.

**Further Resources**

See *The Health Foundation. Evaluation: what to consider. 2015* 60 for a list of websites, articles, webinars and other guidance on various aspects of impact evaluation, which may help locate further information for the planning, interpretation, and development of a successful impact evaluation.^5 23 55^

**Education into practice**

- What interventions have you designed or experienced aimed at transforming your service? Have they been evaluated?
- What types of routine data are collected about the care you deliver? Do you know how to access them and use them to evaluate care delivery?
- What resources are available to you to support impact evaluations for interventions?

## Notes

**Contributors** GMC, SC, ATW and AS designed the structure of the report. GMC wrote the first draft of the manuscript. SC wrote table 2. ATW wrote table 3. AS and GMC critically revised the manuscript for important intellectual content. All authors approved the final version of the manuscript.

Provenance and peer review: This article is part of a series commissioned by The BMJ based on ideas generated by a joint editorial group with members from the Health Foundation and The BMJ, including a patient/carer. The BMJ retained full editorial control over external peer review, editing, and publication. Open access fees and The BMJ’s quality improvement editor post are funded by the Health Foundation.

Patient and/or members of the public were not involved in the creation of this article.
